# Ethanol Extract of Leaves of *Cassia siamea Lam* Protects against Diabetes-Induced Insulin Resistance, Hepatic, and Endothelial Dysfunctions in *ob/ob* Mice

**DOI:** 10.1155/2019/6560498

**Published:** 2019-09-17

**Authors:** Camille Koffi, Raffaella Soleti, Mathieu Nitiema, Patricia Mallegol, Gregory Hilairet, Julien Chaigneau, Jerome Boursier, Mamadou Kamagate, Soazig Le Lay, Henri Maxime Die-Kakou, Ramaroson Andriantsitohaina

**Affiliations:** ^1^Laboratory of Clinical Pharmacology, Université Félix Houphouët-Boigny, Côte d'Ivoire; ^2^INSERM UMR1063, Stress Oxydant et Pathologies Métaboliques, Faculté de Santé, Université d'Angers, Université Bretagne Loire, Angers, France; ^3^EA 3859, Hémodynamique, Interaction Fibrose et Invasivité Tumorales Hépatiques (HIFIH), 49 933 Angers, France; ^4^Laboratory of Clinical Pharmacology, University of Alassane Ouattara, Côte d'Ivoire

## Abstract

Despite long traditional utilization and some reports on the antihyperglycemic and antihyperlipidemic action of *Cassia siamea*, the mechanisms involved have not been investigated yet. Thus, the objective of the present study was to investigate whether and how oral administration of the ethanolic extract of *Cassia siamea Lam* leaves (LECS) improves glucose and insulin homoeostasis, liver damage, and endothelial dysfunction in an experimental model of type 2 diabetes, the leptin-deficient *ob/ob* mice. Oxidative stress and protein expression of insulin-dependent and insulin -independent signaling pathways were studied. Obese **(***ob/ob*) vs. control (*ob/+*) mice were treated daily with intragastric administration of either vehicle or LECS (200 mg/kg, per day) for 4 weeks. Fasting blood glucose, body weight, food intake, glucose and insulin tolerance, oxidative stress, and liver damage as well as vascular complications with respect to endothelial dysfunction were examined. Administration of LECS in obese mice significantly reduced blood glucose and insulin levels, improved glucose tolerance and insulin sensitivity, and restored the increase of circulating AST and ALT without modification of body weight and food intake. These effects were associated with increased activity of both insulin and AMPK pathways in the liver and skeletal muscles. Of particular interest, administration of LECS in obese mice completely prevented the endothelial dysfunction resulting from an increased NO^·^ and decreased reactive oxygen species (ROS) production in the aorta. Altogether, oral administration of LECS remarkably attenuates features of type 2 diabetes on glucose, hepatic inflammation, insulin resistance, endothelial function, and vascular oxidative stress, being as most of these effects are related to insulin-dependent and insulin-independent mechanisms. Therefore, this study points for the therapeutic potential of *Cassia siamea* in correcting both metabolic and vascular alterations linked to type 2 diabetes.

## 1. Introduction

Type 2 diabetes is highly prevalent and is one of the leading causes of mortality and morbidity worldwide, most often associated with obesity. Indeed, it affects over 400 million adults, and the number is expected to rise to 550 million by the year 2030 [1]. Type 2 diabetes is a multifactorial disease involving genetic and environmental factors [2]. The pathophysiological changes are characterized by *β*-cell dysfunction, insulin resistance in the liver, and skeletal muscle and chronic inflammation, all of which hamper control of blood glucose levels and contribute to the development of vascular complications. Traditional antihyperglycemic drugs have helped to improve glycemic control without reducing cardiovascular complications. Pharmacological and life style interventions should address all potential risk factors to improve the cardiovascular outcomes in patients with type 2 diabetes [3].

It is generally accepted that medicinal plants can have health-promoting properties [4] and can be therapeutic alternatives in the management of type 2 diabetes [5]. This strategy involves exploitation of the potential medicinal plants from the local pharmacopoeia. In this context, several studies have shown that plant extracts are veritable sources of antidiabetic compounds [6]. For example, Madeglucyl and metformin, both oral antidiabetic drugs, are isolated from *Eugenia jambolana* (Rutaceae) and *Galega officinalis* (Fabaceae), respectively [7, 8]. The mechanisms of natural products for glucose control in diabetes include the inhibition of glucose absorption (via inhibition of the glucose transporter (GLUT)), improvement of insulin sensitivity, protection of *β*-cell damage, increase of insulin release, enhancement of antioxidant defense, attenuation of inflammation, and modulation of carbohydrate metabolism pathway. These effects are under the regulation of insulin-dependent (via insulin receptor, IR_*β*_, and Akt) and insulin-independent (via adenosine monophosphate-activated protein kinase (AMPK)) signaling pathways [9, 10].

*Cassia siamea Lam* (Fabaceae), a folklore medicinal plant, is known for its therapeutic potential in diabetes and hypertension [11]. Phytochemical screening of this plant reveals the presence of polyphenols, flavonoids, isoflavonoids, phenolic acids, triterpenoids, chromones, anthraquinones, bianthraquinones, sennosides, steroids, and carotenoids [10]. Previous pharmacological studies show that organic and aqueous extracts of *Cassia siamea* possess high antioxidant potential [12], reduce hyperglycemia by 39.38–54.32%, improve lipid profile in alloxan-induced diabetes rats [13, 14], and induce vasodilatation in isolated rat mesenteric arteries [15]. Cassiamin A, isolated from the ethyl acetate extract of *Cassia siamea*, exhibits pancreatic lipase inhibitory activity that participates in its antiobesity effect [16].

This study was undertaken to investigate the effects of the antidiabetic potential of the leaf ethanol extract of *Cassia siamea Lam* (LECS) that takes into account glucose control *via* insulin-dependent and insulin-independent pathways on the hepatic damage and inflammation and vascular defects, namely, endothelial dysfunction in an experimental model of type 2 diabetes, i.e., the leptin-deficient *ob/ob* mice.

## 2. Materials and Methods

### 2.1. Plant Material

The fresh leaves of *Cassia siamea* (ITIS No. 505177) were collected in Adiopodoumé (Ivory Coast), in June 2015. This plant has been authenticated by Doctor Assi Yapo, a botanist in the Department of Biosciences at the Centre National de Floristique (Université Félix Houphouët-Boigny, Abidjan, Ivory Coast), and the voucher specimen was registered under No. 126/97.

### 2.2. Extraction Procedure

Ethanol extract was prepared as previously described [13]. Briefly, the fresh leaves of *C. siamea* were washed, dried, pulverized, and extracted by cold maceration in ethanol (80%) for 48 h. Then, the extract was filtered, distilled off at 40°C in a circulating air oven (Memmert®, Germany) to obtain a powder LECS, and stored at 4°C until use. The extraction ratio expressed as LECS mass/powder of leaf mass was 0.14.

### 2.3. Ethics Statement

Present study was conducted in accordance with the guidelines and authorization with French Ministry of Agriculture regulations based on the European Community. Upon local ethics committee approval, the animal protocol followed in the present study was authorized by the French Ministry of National Education, Higher Education and Research (APAFIS#265-2015110916015679 v2).

### 2.4. Animals

Three- to four-month-old *ob/ob* or *ob/+* male and female mice were obtained from the animal housing unit of the University of Angers (Angers, France) and maintained under an environmentally controlled facility (temperature 22°C and 12-hour light and dark cycles) with free access to food and water. The animals were acclimatized for 7 days prior to the experiments and provided rodent chow and water *ad libitum*. The leptin genotypes of the animals were verified by the PCR method at the laboratory.

### 2.5. Pharmacological Study

#### 2.5.1. Treatment

Four groups of animals have been constituted: two groups of obese mice (*n* = 8 mice for each group) and two groups of controls (*n* = 8 mice for each group). Obese mice and their wild type were treated daily for 28 days by intragastric gavage either with vehicle (2% Tween 80) or with LECS (200 mg/kg, per day). The dose 200 mg/kg/day of LECS was chosen as the most effective in exhibiting antihyperglycemic properties in an experimental model of a diabetic rat [13].

#### 2.5.2. Food Intake, Body Weight, and Fasting Blood Glucose

Food intake and body weight gain of all mice were monitored during the 28-day experimental period. At an interval of 7 days, blood samples were collected from the tail vein of the mice after overnight fasting for the estimation of the blood glucose level using a glucometer (Freestyle Seed Optium®, Australia) and compatible blood glucose test strips.

#### 2.5.3. Glucose Tolerance Test (GTT) and Insulin Tolerance Test (ITT)

After twenty-one days of treatment, mice were fasted overnight for GTT. Fasting glycemia was measured and defined as time zero (0 min). Then, all animal groups received an intraperitoneal (i.p.) load of (+) D-glucose (1.0 g/kg). Tail vein blood samples were withdrawn 10, 20, 30, 60, 90, and 120 min after the administration of glucose. Blood glucose was monitored using a glucometer.

ITT was performed on the twenty-fifth day of treatment. Tail blood was collected before (0 min) and at 15, 30, 45, and 60 min after i.p. injection of insulin (0.75 U/kg).

#### 2.5.4. Sample Collection

At the end of the experimental protocol, mice were euthanized by gradient CO_2_ inhalation. The liver and soleus skeletal muscle were isolated, weighed, immediately placed in liquid nitrogen, and stored at -80°C until further use, while the thoracic aorta was isolated for vascular reactivity assessment.

#### 2.5.5. Biochemical Analysis

Blood samples were centrifuged at 900 g for 10 min to obtain plasma, which was stored at -20°C until the following parameters had been determined: glucose, aspartate aminotransferase (AST) and alanine aminotransferase (ALT), triglycerides, cholesterol, HDL, and LDL using a Konelab™ 20 Clinical Chemistry Analyzer (Thermo Scientific™, Waltham, MA). Plasma insulin was determined by ELISA (Millipore, Germany).

#### 2.5.6. Western Blotting

Tissue samples from the liver and muscle were frozen in liquid nitrogen and homogenized in a buffer containing 500 *μ*L sodium dodecyl sulfate (SDS, 20%), 100 *μ*L sodium orthovanadate, 50 *μ*L Na pyrophosphate, 200 *μ*L Tris HCl 500 mM (pH 7.4), and 400 *μ*L antiprotease. The tissue suspensions are centrifuged at 15000 g for 15 min at 4° C. Supernatants containing the proteins were collected and stored at -80° C until use. Forty *μ*g of proteins from tissue lysates were run on 10% reducing SDS-acrylamide gels (Invitrogen, Carlsbad, CA). The samples were electrotransferred to nitrocellulose membranes, and the membranes were then saturated at room temperature for 1 h in TBS-T (20 mM Tris base, 61.5 mM NaCl pH 7.8, and 0.1% Tween 20) buffer containing 5% bovine serum albumin (BSA). After washing, the membranes were incubated with a primary antibody for 2 h and then incubated for 90 min either with horseradish peroxidase-secondary anti-rabbit or with anti-mouse antibodies (USBiological, Salem, MA). The blots were visualized using the enhanced chemiluminescence system (ECL Plus, Amersham Biosciences, Piscataway, NJ) and the SuperSignal West Femto (Thermo Scientific Pierce, Brebières, France) and quantified by densitometry. The primary antibodies used were polyclonal anti-phospho-Akt (Ser 473) (SAB, College Park, MD), polyclonal anti-Akt (Cell Signaling Technology, Danvers, MA), polyclonal anti-phospho-AMPK*α* (Thr172) (Cell Signaling Technology), polyclonal anti-AMPK*α* (Cell Signaling Technology), polyclonal anti-phospho-insulin receptor *β* (Y1162/1163) (Santa Cruz Biotechnology, Santa Cruz, CA), polyclonal anti-insulin receptor *β* (Santa Cruz Biotechnology), polyclonal anti-GLUT4 (Santa Cruz Biotechnology), polyclonal anti-GLUT2 (Santa Cruz Biotechnology), and polyclonal anti-*β*-actin (Sigma-Aldrich, St. Louis, MO).

#### 2.5.7. Nitric Oxide (NO^·^) and Reactive Oxygen Species (ROS) Evaluation

Detection of NO^·^ and ROS productions in mice femoral artery rings was performed using a previously described electronic paramagnetic resonance technique (Spectrometer MiniScope MS200, Magnettech, Berlin, Germany) [17]. Fe^2+^ diethyldithiocarbamate (DETC, Sigma-Aldrich) was used as a spin trap for NO^·^, whereas ROS detection was performed using deferoxamine-chelated Krebs-HEPES solution containing 1-hydroxy-3-methoxycarbonyl-2,2,5,5-tetramethylpyrrolidine (CMH 500 *μ*mol/L, Noxygen, Mainz, Germany), deferoxamine (25 *μ*mol/L, Sigma-Aldrich), and DETC (5 *μ*mol/L, Sigma-Aldrich).

#### 2.5.8. Ex Vivo Vascular Reactivity

After 28 days of treatment, the aorta from control and obese mice was dissected and placed in modified Krebs-Henseleit bicarbonate solution composed of NaCl (130 mM), NaHCO_3_ (14.9 mM), KCl (3.7 mM), KH_2_PO_4_ (1.2 mM), MgSO_4_ (1.2 mM), (+) D-glucose (11 mM), and CaCl_2_ (1.6 mM). Then, aortic rings were mounted on a wire myograph (Danish Myo Technology, Aarhus, Denmark) as previously described [18] containing 7 mL oxygenated (95% O_2_, 5% CO_2_) Krebs-Henseleit solution pH 7.4 and maintained at 37°C. The functionality of the endothelium was assessed by the ability of acetylcholine (Ach, Sigma-Aldrich) to induce relaxation. Thus, cumulative addition of Ach (10^−9^-10^−5^ M) was assessed on a vessel precontracted with the thromboxane analogue (U-46619, Sigma-Aldrich) at 80% of its maximal contraction. Concentration-response curves produced by Ach in aortic rings were expressed as a *percentage* of the precontraction by U-46619.

In addition, in another set of experiments, the effect of LECS on vasorelaxation was assessed in aortic rings from wild-type mice and the EC_80_ (500 *μ*g/mL) was calculated. Then, the implication of endothelium-derived relaxing factors, in response to LECS response such as NO^·^ and prostacyclin, were evaluated using specific NO-synthase and cyclooxygenase (COX) inhibitors, L-N^G^-Nitroarginine Methyl Ester (L-NAME, 10^−4^ M) and indomethacin (10^−5^ M), respectively.

After that, LECS pretreatment on insulin vasorelaxation action was evaluated. Indeed, aortic rings from wild-type mice were isolated and incubated with 500 *μ*g/mL LECS for 30 min and then precontracted with U-46619 (10^−8^ M). When the contraction reached a steady state, increasing concentrations of insulin (10^−8^–3.10^−5^ M) were added cumulatively to produce relaxation. The relaxation produced by insulin was expressed as a percentage of the U-46619-induced contraction.

### 2.6. Statistical Analysis

Statistical analysis was performed using GraphPad Prism 5.1® software. Data were expressed as mean ± SEM of five to eight replicates and subjected to one-way or two-way analysis of variance (ANOVA) followed, when necessary, by Turkey's test or the Bonferroni posttest to determine significant differences in all the parameters. Values were considered statistically significant at *p* < 0.05.

## 3. Results

### 3.1. LECS Does Not Affect Body Weight and Food Intake in Obese Mice

**Figure 1:**
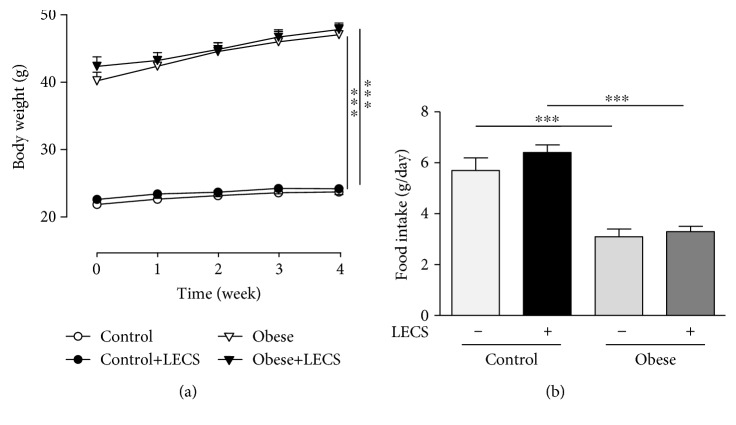
LECS does not affect body weight gain and food intake of *ob/ob* mice. Effects of LECS on body weight (a) and food intake (b) after 4-week administration in *ob/ob* and control mice. The results are expressed as mean ± S.E.M. (*n* = 8/group; ^∗∗∗^*p* < 0.001 vs. lean mice).

Figure 1 shows that *ob/ob* mice displayed a greater body weight and lower food intake compared to lean mice. LECS did not significantly modify the body weight and the food intake in the two groups of mice.

### 3.2. LECS Does Not Affect Plasma Lipid Levels

LECS treatment did not modify triglyceride, cholesterol, HDL, and LDL levels, independently from the strain (Table 1).

### 3.3. LECS Exhibits Antihyperglycemic Effects and Improves Glucose and Insulin Tolerance in Obese Mice

**Figure 2:**
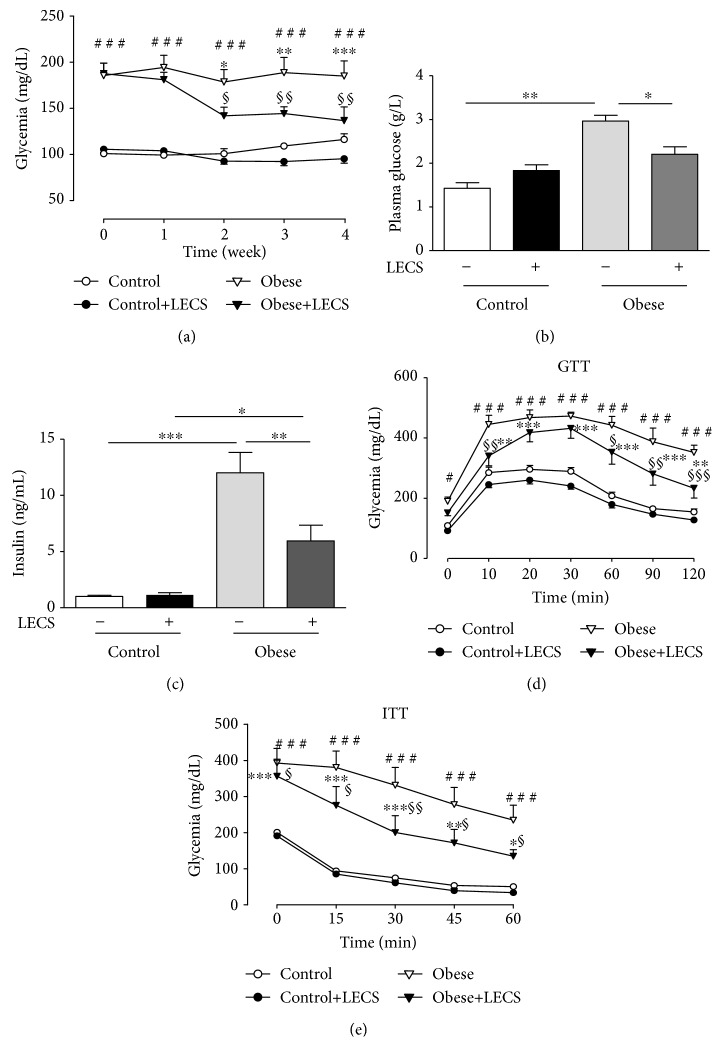
LECS exhibits antihyperglycemic effects and improves glucose and insulin tolerance of *ob/ob* mice. Effects of LECS on fasting blood glucose (a), plasma glucose (b), insulin (c), glucose (d), and insulin (e) tolerance, after intraperitoneal (i.p.) administration either of D-glucose (1.0 g/kg) or insulin (0.75 UI/kg). The results are expressed as mean ± S.E.M. (*n* = 8/group; ^#^*p* < 0.05, ^##^*p* < 0.01, and ^###^*p* < 0.001 vs. control; ^∗^*p* < 0.05, ^∗∗^*p* < 0.01, and ^∗∗∗^*p* < 0.001 vs. control+LECS; ^∗^*p* < 0.05, ^∗∗^*p* < 0.01, and ^∗∗∗^*p* < 0.001 vs. control+LECS; ^§^*p* < 0.05, ^§§^*p* < 0.01, and ^§§§^*p* < 0.001 vs. obese).

Obese mice displayed a greater circulating glucose level compared to control mice (Figure 2). LECS (200 mg/kg) treatment did not modify the glycemia of lean mice. Interestingly, LECS reduced significantly hyperglycemia in a time-dependent manner in *ob/ob* mice (*p* < 0.01), the effect being maximal after the 2-week treatment (Figure 2(a)). Thus, at the end of treatment, LECS significantly reduced the level glucose of *ob/ob* mice (*p* < 0.05) towards that of control mice (Figure 2(b)). Also, obese mice exhibited a greater circulating insulin level compared to control mice (Figure 2(c)). LECS treatment did not modify the insulin level in lean mice but reduced that of *ob/ob* mice.

The GTT revealed that, after receiving the glucose overload, *ob/ob* mice developed higher values of glycemia than the control animal group throughout the assay (*p* < 0.001, Figure 2(d)). Interestingly, LECS significantly decreased the glucose overload in *ob/ob* mice but not in control animals. The ITT showed that, after receiving insulin injection, the decrease of blood glucose levels was significantly greater after LECS treatment in *ob/ob* mice but not in control animals (Figure 1(e)). These results indicate that LECS improves glucose and insulin intolerance in diabetic *ob/ob* mice.

### 3.4. LECS Improves Hepatic Parameters in Obese Mice

**Figure 3:**
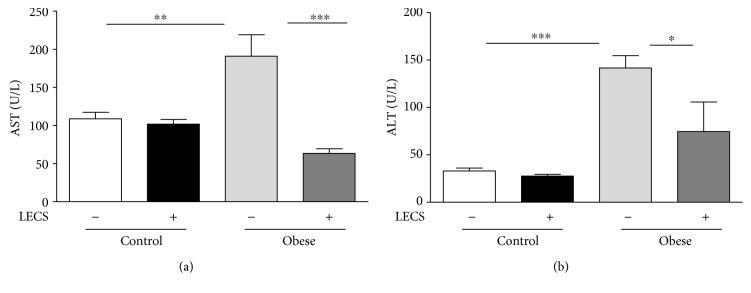
LECS improves hepatic parameters in diabetic *ob/ob* mice. Effects of LECS on hepatic function parameters in lean and *ob/ob* mice: (a) aspartate aminotransferase (AST); (b) alanine aminotransferase (ALT). The results are expressed as mean ± S.E.M. (*n* = 8/group; ^∗^*p* < 0.05, ^∗∗^*p* < 0.01, and ^∗∗∗^*p* < 0.001).

The untreated *ob/ob* mice exhibited significant increase (*p* < 0.05) in plasma activities of ALT and AST when compared with those of control mice (Figure 3). LECS treatment in *ob/ob* mice restored the increases of ALT and AST toward those from control mice suggesting that the extract corrects liver dysfunction.

### 3.5. LECS Beneficial Effects in the Liver and Skeletal Muscle in Obese Mice

**Figure 4:**
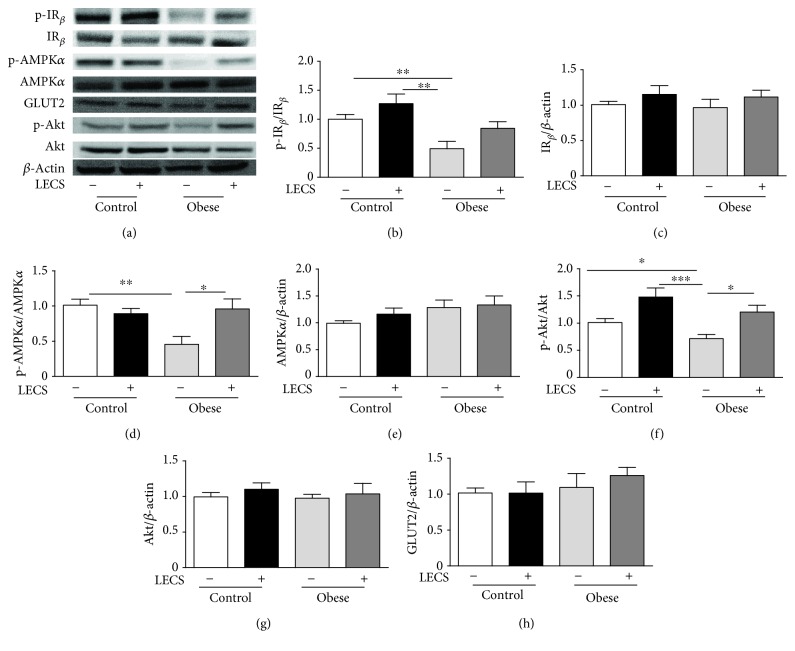
LECS upregulates Akt and AMPK*α* pathways in the liver of diabetic *ob/ob* mice. (a) Representative images of blots. Quantitative evaluation of the protein activation and/or expression in the liver: (b) p-IR_*β*_, (c) IR_*β*_, (d) p-AMPK*α*, (e) AMPK*α*, (f) p-Akt, (g) Akt, and (h) GLUT2. The results are expressed as mean ± S.E.M. (*n* = 8/group; ^∗^*p* < 0.05, ^∗∗^*p* < 0.01, and ^∗∗∗^*p* < 0.001.

**Figure 5:**
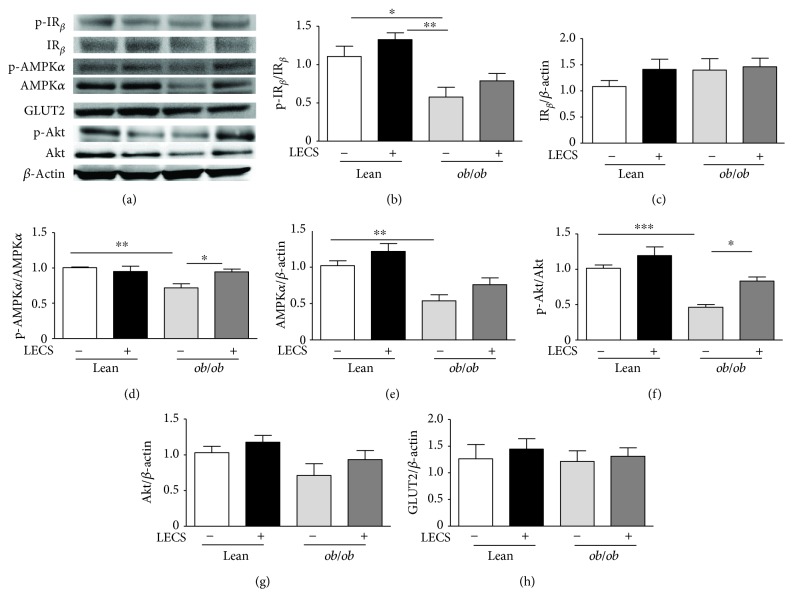
LECS upregulates Akt and AMPK*α* pathways in the skeletal muscle of diabetic *ob/ob* mice. (a) Representative images of blots. Quantitative evaluation of the protein activation and/or expression in the skeletal muscle: (b) p-IR_*β*_, (c) IR_*β*_, (d) p-AMPK*α*, (e) AMPK*α*, (f) p-Akt, (g) Akt, and (h) GLUT4. The results are expressed as mean ± S.E.M. (*n* = 8/group; ^∗^*p* < 0.05, ^∗∗^*p* < 0.01, and ^∗∗∗^*p* < 0.001).

The activation/expression of proteins involved in intracellular glucose utilization pathways, such as IR_*β*_, Akt, AMPK, and GLUTs in the liver and skeletal muscle of *ob/ob* versus control mice, was performed (Figures 4 and 5).

Semiquantitative analysis of IR_*β*_, Akt, AMPK, GLUT2, and GLUT4 showed that their expressions were not significantly altered either in tissues from *ob/ob* mice or in those treated by LECS when compared to that of control mice. Interestingly, the phosphorylation isoform of AKT and AMPK that was reduced in *ob/ob* mice was significantly enhanced by LECS treatment. Taken together, these findings suggest that LECS improved both insulin and AMPK signaling in skeletal and liver tissues from *ob/ob* mice.

### 3.6. LECS Prevents Endothelial Dysfunction

**Figure 6:**
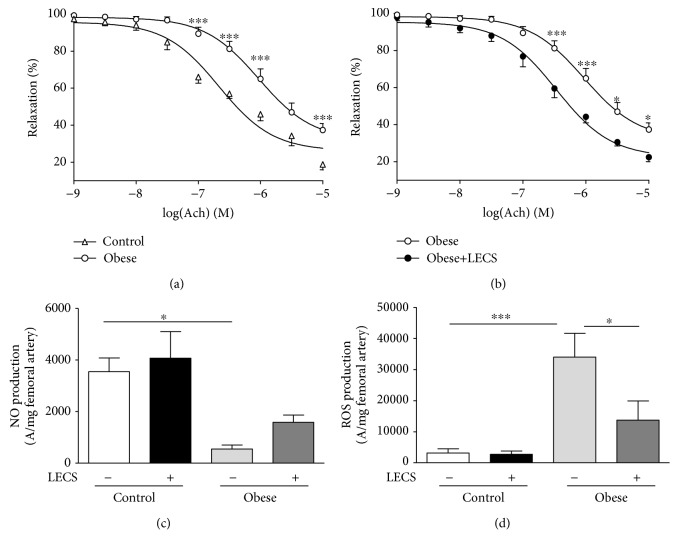
LECS induces vascular endothelial protective activities. Concentration-response curves to Ach in (a) lean and (b) *ob/ob* mice. (c) NO^·^ and (d) ROS productions in the femoral artery. The results are expressed as mean ± S.E.M. (*n* = 8/group; ^∗^*p* < 0.05 and ^∗∗∗^*p* < 0.001).

As expected, the endothelium-dependent relaxation in response to Ach was significantly impaired in the aorta taken from *ob/ob* mice compared to control mice (Figure 6(a)). Remarkably, LECS treatment significantly potentiated the endothelium-dependent relaxation to Ach in the aorta from *ob/ob* mice (Figure 6(b)) but not from control mice (not shown). Thus, Ach response of aortic *ob/ob* mice treated with LECS was reverted toward that of the aorta from control mice. The impaired relaxation to Ach in *ob/ob* mice was associated with a significant decrease of NO^·^ and increase of ROS productions in the femoral artery (Figures 6(c) and 6(d)). LECS had no effect on either NO^·^ or ROS in the femoral artery from control mice. Interestingly, LECS significantly inhibited the increase of ROS in the femoral artery from *ob/ob* mice.

### 3.7. LECS Induces Endothelium-Dependent and Endothelium-Independent Relaxation

**Figure 7:**
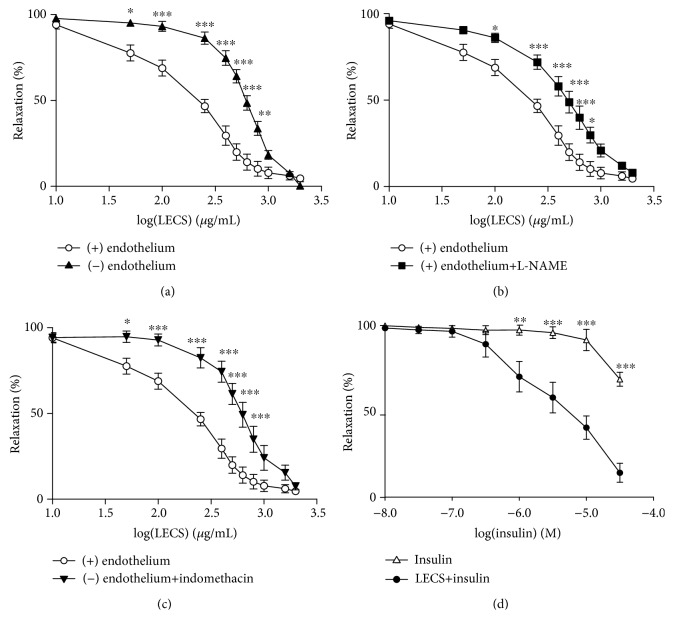
LECS induces endothelium-dependent and endothelium-independent relaxation. Concentration-response curves for LECS-induced relaxation in wild-type aortic rings with and without endothelium (a) and with endothelium in the presence of either L-NAME (10^−4^ M) (b) or indomethacin (10^−5^ M) (c), and effects of LECS on insulin vasorelaxation action (d). The results are expressed as mean ± S.E.M. (*n* = 5/group; ^∗^*p* < 0.05, ^∗∗^*p* < 0.01, and ^∗∗∗^*p* < 0.001).

LECS (10-2000 *μ*g/mL) induced a concentration-dependent relaxation of aortic rings either in the presence or in the absence of a functional endothelium (Figure 7). However, LECS was more efficient in the presence of an endothelium compared to an endothelium-denuded aorta as shown by a significant rightward shift of the concentration-response curve to the compound in the endothelium-denuded aorta (Figure 7(a)). Also, LECS relaxation curves were significantly shifted to the right in the presence of either the NO-synthase inhibitor, L-NAME, or the COX-inhibitor, indomethacin (Figures 7(b) and 7(c)). Besides, preincubation of the aortic rings with LECS (500 *μ*g/mL) significantly potentiated the endothelium-dependent relaxation to insulin (Additional file 2d).

## 4. Discussion

The present study provides evidence that in an experimental model of severe obesity, LECS reduced blood glucose and insulin levels, improved glucose tolerance and insulin sensitivity, and increased activity of hepatic transaminases without modification of body weight and food intake. Accordingly, the improvement of the insulin sensitivity was accompanied by increase of both insulin and AMPK pathways in the liver and skeletal muscle. Additionally, LECS completely prevented the endothelial dysfunction resulting from an increased NO^·^ and decreased ROS production. Thus, LECS not only improves glycemic control and insulin resistance but also improves the endothelial function of *ob/ob* mice.

Indeed, based on the experimental model used in the present study, the *ob/ob* mouse develops obesity as early as 1 month of age with progressively increasing food intake, body weight, glycemia, insulin levels, insulin resistance, and pancreatic, hepatic, and endothelial dysfunction through leptin deficiency in the hypothalamus [19].

It is interesting to note that oral administration of LECS, at the dosage used, did not significantly decrease body weight and daily food intake in *ob/ob* mice. Thus, LECS did not induce toxicity or orexigenic effects under the experimental condition used. These results are in accordance with previous studies with traditional consumption of this plant. In line with these data, young fruits and leaves of *Cassia siamea* are consumed as vegetables in Thailand and India [20], without obvious orexigenic and toxic actions [11].

The most important finding of the present study was the amelioration of the diabetic-related glucose tolerance and insulin resistance state by the LECS treatment, as shown by the restoration of hyperglycemia and reduction of insulinemia without hypoglycemic or hypoinsulinemic effect in lean mice and the increased insulin sensitivity. Thus, daily feeding of mice for 4 weeks with LECS (200 mg/kg) is adequate to produce a sufficient circulating concentration of compounds to induce metabolic and vascular effects. In our previous study, LECS exhibits an antihyperglycemic effect in nondiabetic rats after induction of hyperglycemia with 2 g/kg of glucose feeding within 1-5 h [21]. In addition, oral administration of leaf methanolic extract (250, 500 mg/kg) for 3 weeks decreases high blood glucose levels in streptozotocin diabetic rats [14].

The sequence of events leading to the development of insulin resistance in diabetes is incompletely understood. Because of the central role of the liver and skeletal muscle in the whole-body energy homeostasis, both the liver and the skeletal muscle insulin sensitivity appear to be crucial in the development of this state. In the present study, we found that LECS increased the expression of phosphorylated IR_*β*_, Akt, and AMPK, which were inhibited in *ob/ob* mice. Thus, it is likely that LECS activates signaling pathways known to improve insulin sensitivity including insulin receptor substrate-1, phosphatidylinositol-3-kinase, protein kinase B (PKB/Akt), and AMPK phosphorylation. Although we did not find any change in GLUT2 or GLUT4 expressions, activation of these signaling pathways is known to promote translocation of GLUT2 in the liver and GLUT4 in the muscle. Such a mechanism might be implicated in the ability of LECS to prevent the development of an insulin resistant state observed in *ob/ob* mice in the present study.

Progression of chronic diabetes in *ob/ob* mice is also associated with the development of hyperlipemia in addition to insulin resistance [22]. Hyperlipemia is characterized by the increase of serum free fatty acid, triglyceride, and total cholesterol [23]. Previously, we reported that organic extracts of *Cassia siamea* display potential antiobesity compounds via its inhibitory activity on the pancreatic lipase. Such a mechanism may also play a role in the antidiabetic property of LECS although it did not modify plasma lipid levels in the present study.

Chronic mild elevations of hepatic transaminases, such as ALT and AST, are frequently found in type 2 diabetes [24]. The underlying mechanism of diabetes that contributes to liver damage is the combination of increased oxidative stress and an aberrant inflammatory response, which activates the transcription of proapoptotic genes and damages hepatocytes [25]. Interestingly, LECS treatment normalized the levels of AST and ALT in the present study. The high antioxidant potency of *Cassia siamea* may be responsible at least in part for its hepatoprotective activity [12].

Endothelial dysfunction has been described to be inextricably associated with obesity and insulin resistance [26]. Indeed, insulin receptors are present on endothelial cells, and the disruption of endothelial insulin signaling may affect endothelial function [27]. At physiological concentrations, insulin stimulates both endothelial NO-synthase activity and expression in endothelial cells and promotes endothelium-dependent relaxation [28, 29]. The phosphorylation of eNOS at Ser 1177 can be blunted by hyperglycemia [30] and elevated concentrations of saturated free fatty acid under conditions of vascular insulin resistance. Most importantly, we found that LECS completely restored the endothelial dysfunction in *ob/ob* mice, probably due to enhanced insulin sensitivity as discussed above. The impaired relaxation to Ach in *ob/ob* mice was associated with a significant decrease of NO^·^ and an increase of ROS productions in the femoral artery. Interestingly, LECS significantly inhibited the increase of ROS in the femoral artery from *ob/ob* mice. Indeed, various extracts of *Cassia siamea* have been reported to possess high antioxidant potential [11]. In line with the *in vivo* effects, LECS promotes endothelium-dependent relaxation via NO-synthase and COX inhibitor-sensitive mechanisms and potentiates insulin-induced relaxation *ex vivo* in control mice. Thus, LECS itself can act on the endothelium with special emphasis on endothelial NO-synthase activation and NO^·^ release. Together, these data may suggest that an increased antioxidant defense mechanism occurs to reduce oxidative stress in these animals and therefore participates in the restoration of endothelial function and NO^·^ bioavailability in conjunction with the direct effect of LECS on the endothelium with respect to NO and COX-vasodilatory metabolites. All these effects concur with the protective effects of LECS on endothelial dysfunction. The latter may also participate in the correction of metabolic features of this experimental model of diabetes with severe obesity.

The identification of the exact mechanism by which LECS reduced insulinemia has not been assessed and represented a limitation of the present study. Nevertheless, we have reviewed previously that LECS contains lupeol, D-pinitol, luteolin, and some dihydronaphtalenone. These compounds might be implicated in the *in vivo* effect of LECS and, hence, might act on the tissues regulating insulin resistance such as the liver and skeletal muscle and also the endothelium *in vivo*. Further studies are needed to identify the compounds that support the LECS effects.

## 5. Conclusion

The present study provides scientific basis and evidence for the safe use of *Cassia siamea* by traditional healers on the preventive/curative purposes for diabetic symptoms. Thus, LECS concomitantly corrected the metabolic and endothelial alterations in an experimental model of diabetes. Improved glucose tolerance, insulin sensitivity, liver function, and endothelial function participate to these beneficial effects of LECS probably *via* activation of insulin and AMPK pathways. This study supports the hypothesis that *Cassia siamea* may be therapeutically relevant in reducing metabolic and cardiovascular risks in patients with type 2 diabetes.

## Figures and Tables

**Table 1:** Effect of LECS on plasma lipid levels.

	Control	Control+LECS	Obese	Obese+LECS
Triglycerides (g/L)	0.57 ± 0.04	0.53 ± 0.05	0.53 ± 0.06	0.56 ± 0.08
Cholesterol (g/L)	1.1 ± 0.1	0.9 ± 0.05	1.3 ± 0.1	1.3 ± 0.2
HDL (g/L)	0.79 ± 0.06	0.86 ± 0.07	1.09 ± 0.1	1.07 ± 0.13
LDL (g/L)	0.13 ± 0.01	0.1 ± 0.02	0.15 ± 0.02	0.17 ± 0.03

Triglycerides, cholesterol, HDL, and LDL plasma concentration in control and obese mice treated or not with LECS. The results are expressed as mean ± SEM.

## Data Availability

The data used to support the findings of this study are included within the article.
